# Cardiac Rhythm Conversions and the Outcome in Refractory Out-of-Hospital Cardiac Arrest: Extracorporeal Versus Conventional Resuscitation

**DOI:** 10.1097/CCM.0000000000006787

**Published:** 2025-07-16

**Authors:** Stepan Havranek, Jakub Neuhöfer, Michaela Vesela, Petra Kavalkova, Daniel Rob, Zdenka Fingrova, Jana Smalcova, Ondrej Franek, Michal Huptych, Milan Dusik, Jan Pudil, Vojtech Weiss, Ales Linhart, Jan Belohlavek

**Affiliations:** 1 2nd Department of Medicine—Department of Cardiovascular Medicine, First Faculty of Medicine, Charles University and General University Hospital in Prague, Prague, Czech Republic.; 2 Emergency Medical Service Prague, Prague, Czech Republic.; 3 Czech Institute of Informatics, Robotics and Cybernetics (CIIRC), Czech Technical University in Prague, Prague, Czech Republic.

**Keywords:** cardiopulmonary resuscitation, extracorporeal cardiopulmonary resuscitation, extracorporeal membrane oxygenation, heart rhythm, refractory cardiac arrest

## Abstract

**OBJECTIVES:**

A Prague out-of-hospital cardiac arrest (OHCA) study has demonstrated that an invasive approach (early transport to the hospital, extracorporeal cardiopulmonary resuscitation [ECPR]) is a feasible and effective treatment strategy in refractory OHCA. This post hoc analysis of the Prague OHCA study aimed to stratify the prognosis of patients according to the detailed course of heart rhythm during prehospital and early hospital periods.

**DESIGN, SETTING, AND PATIENTS:**

This analysis included all 256 patients (median age 58, 17% females) randomized to the Prague OHCA study. The sequence of heart rhythms during the prehospital period was analyzed in terms of neurologic outcomes after 180 days. The primary outcome was a composite of survival with Cerebral Performance Category 1 or 2 at 180 days.

**INTERVENTIONS:**

None.

**MEASUREMENTS AND MAIN RESULTS:**

Within the study cohort, 156 (61%) manifested ventricular fibrillation (VF), 45 pulseless electrical activity, and 55 asystole as the initial rhythm. Patients with an initial VF who reached a sustained recovery of spontaneous circulation (ROSC) had the highest proportion of reaching a primary outcome (32/44 [73%]). Patients who had one or more episodes of asystole during cardiopulmonary resuscitation had the lowest rate of primary endpoint (5/39 [13%]). Patients who experienced intermittent ROSC showed a higher success rate in achieving the primary outcome when treated with an invasive-based approach (including ECPR) compared with the conventional strategy (26/34 [76%] vs. 24/50 [48%]; *p* < 0.05).

**CONCLUSIONS:**

Achieving ROSC is the best prognostic marker in OHCA patients with an initially refractory VF. Patients with intermittent ROSC after the initial VF and ongoing VF seem to be optimal candidates for an invasive approach. Asystole detection at any time during resuscitation is a strong negative prognostic marker, irrespective of the initial rhythm.

**KEY POINTS**

**Question**: The prognostic role of rhythm deterioration during refractory out-of-hospital cardiac arrest (OHCA) has not been fully described.

**Findings**: The post hoc analysis of the Prague OHCA trial identified the role of rhythm conversion on patient outcome. The most favorable predictor in patients with refractory OHCA was intermittent or sustained recovery of spontaneous circulation after initial ventricular fibrillation. Asystole at any point during resuscitation strongly indicates a poor prognosis, regardless of the initial rhythm or treatment strategy.

**Meaning**: Patients with intermittent recovery of spontaneous circulation are ideal candidates for invasive treatment, including extracorporeal cardiopulmonary resuscitation.

Out-of-hospital cardiac arrest (OHCA) places a considerable burden on society (1). For patients who fail to achieve a recovery of spontaneous circulation (ROSC), the likelihood of survival after transport to the hospital while undergoing ongoing cardiopulmonary resuscitation (CPR) is minimal, typically below 4% with the use of conventional CPR (CCPR) alone (2, 3).

The temporary replacement of failing circulation by extracorporeal CPR (ECPR) has been recognized as a promising approach in refractory cardiac arrest (CA) (4–8). The Prague OHCA study offers encouraging evidence supporting the use of extracorporeal ECPR in select patients with refractory OHCA (9). While not universally applicable, ECPR has demonstrated the potential to improve survival outcomes in patients who meet specific criteria (10).

Initial cardiac rhythm, response time of bystander CPR, emergency medical services response time, duration, and quality of CPR critically affect the outcome in OHCA (11–14). We have shown previously that outcomes of refractory OHCA with initial rhythm being ventricular fibrillation (VF) are far more favorable than those with initial asystole or pulseless electrical activity (PEA) (15). However, many patients manifest rhythm changes during ongoing CPR (16, 17). Observational studies showed that clinical outcomes could differ in patients with a transient organized rhythm compared with uninterrupted VF or a transient asystole (16, 18–20). Furthermore, changing from a nonshockable to a shockable rhythm decreases the risk of unfavorable outcomes (21–23). Recently, we published data from a prospective registry showing that the rhythm at hospital admission affected ECPR outcomes (24). However, the relationship between the rhythm conversions in refractory OHCA and clinical outcomes has not been fully elucidated. Further, the potential benefits of an ECPR-based approach compared with CCPR concerning different rhythm profiles remain unclear.

Therefore, we hereby present an analysis of the rhythm conversions during CPR and their impact on patient outcomes in refractory OHCA and aim to analyze the exploitability of the ECPR-based vs. conventional approach in a randomized population.

## METHODS

The current study is a post hoc analysis of the Prague OHCA study, a randomized controlled trial comparing an ECPR-based/invasive approach (early transport to hospital under mechanical CPR, ECPR, and immediate invasive assessment and therapy) to standard/conventional treatment in the refractory OHCA population (9, 25). The study was performed according to good clinical practice and in accordance with the ethical standards of the responsible regional committee on human experimentation and with the Helsinki Declaration of 1975. The Prague OHCA study was approved by the Ethics committee of the General University Hospital in Prague (192/11 S-IV, date February 17, 2011).

### Study Population

A detailed protocol of the main study and primary results have been described previously (9, 25). In brief, the study enrolled adults over 18 years old, with a witnessed OHCA of a presumed cardiac etiology, who were given a minimum of 5 minutes of advanced cardiac life support without ROSC and who remained unconscious. The patients were randomized in a 1:1 ratio into two study arms: invasive or standard. The termination of resuscitation efforts followed the valid European Resuscitation Council (ERC) guidelines (9, 25–27).

### Intervention

Patients randomized into the standard/conventional arm were managed on-site by continued advanced cardiac life support. Drug use, further defibrillations, or other interventions followed the available ERC guidelines (26, 27). If ROSC was achieved (defined as an organized cardiac electrical activity with a palpable pulse), transportation to the hospital was initiated for further treatment as per current guidelines.

In the ECPR-based/invasive arm, the immediate intraarrest transfer directly to the cardiac center catheterization laboratory was initiated after randomization. Then, if ROSC was not achieved en route or at admission, ECPR (implantation of venoarterial extracorporeal membrane oxygenation [ECMO]) was performed. The use of drugs, further defibrillations, or other interventions during transportation followed the ERC guidelines (26, 27). Post-resuscitation care was standardized in both study arms.

### Initial Rhythm and Rhythm Change

For the present study, the initial rhythm was defined as the first documented rhythm by the Emergency Medical System. Any prehospital rhythm conversions, either spontaneous or due to defibrillation, were evaluated. The rhythm at each assessment was placed into one of four groups: ROSC, VF, PEA, and asystole. The final rhythm was the last documented cardiac rhythm before withdrawal from the study or the rhythm at admission to the hospital.

### Outcomes

The primary outcome was the composite of a 180-day survival with a favorable neurologic status, defined as no or minimal neurologic impairment (Cerebral Performance Category [CPC], 1 or 2).

### Statistical Analysis

The numeric variables are expressed as medians and interquartile ranges. The two-sided Mann-Whitney, Kruskal-Wallis, and Fisher exact test (for a 2 × 2 table), chi-square test, and analysis of variance were used when appropriate. All the presented *p* values are two-tailed. The Cox regression analysis for prediction of an unfavorable clinical outcome was performed separately for data available during CPR and after the early hospital evaluation (within 1 hr). A *p* value of less than 0.05 was considered as statistically significant. Statistical analyses were performed with MedCalc Statistical Software, Version 19.7 (MedCalc Software, Ostend, Belgium; 2021), RStudio 2022.07.2+576 (RStudio Team [2020]. RStudio: Integrated Development for R. RStudio, PBC, Boston, MA, URL: http://www.rstudio.com/).

## RESULTS

### Baseline Clinical Data, Prehospitalization Phase

During the study period, 256 patients (median age 58 yr, 17% females) were enrolled and analyzed. The baseline demographic and clinical data are described in **Table 1** and **Supplemental Table 1** (https://links.lww.com/CCM/H762). Out of the entire study cohort, 156 patients (61%) manifested VF as the initial rhythm. The rest of the patients had an initially nonshockable rhythm (45 [18%] PEA and 55 [21%] asystole).

**TABLE 1.** Baseline Demographical and Clinical Data

Initial Rhythm	Ventricular Fibrillation (*n* = 156)	Pulseless Electrical Activity (*n* = 45)	Asystole (*n* = 55)	*p*
Age (yr)	56 (45–64)	62 (54–66)	58 (47–69)	0.07
Gender (male)	141 (90%)	31 (69%)	40 (73%)	0.0003
Bystander CPR	154 (99%)	44 (98%)	54 (98%)	0.89
Dispatcher assisted CPR	133 (85%)	24 (53%)	46 (84%)	< 0.0001
Randomized to				
Standard	84 (54%)	24 (53%)	24 (44%)	0.41
Invasive	72 (46%)	21 (47%)	31 (56%)	
Cross-over				
Standard → invasive	10/84	0/24	1/24	0.007
Invasive → standard	2/72	1/21	6/31	
Time of CPR (time to recovery of spontaneous circulation or ECLS) (min)	54 (33–69)	50 (42–68)	56 (37–67)	0.62
Admitted to hospital	136 (87%)	33 (73%)	41 (75%)	0.03
Declared death	33 (21%)	20 (44%)	22 (40%)	0.0015
ECLS implanted	57 (37%)	17 (38%)	18 (33%)	0.85
Cause of cardiac arrest (including autopsy findings)				
Acute coronary syndrome	89 (57%)	15 (33%)	23 (42%)	< 0.0001
Chronic coronary artery disease	29 (19%)	1 (2%)	2 (4%)	
Pulmonary embolism	1 (1%)	15 (33%)	8 (15%)	
Chronic heart failure	8 (5%)	2 (4%)	4 (7%)	
Cardiomyopathy	7 (5%)	1 (2%)	1 (2%)	
Myocarditis	5 (3%)	1 (2%)	2 (4%)	
Aortic stenosis	5 (3%)	1 (2%)	2 (4%)	
Aortic dissection type A	1 (1%)	1 (2%)	2 (4%)	
Intracranial hemorrhage	1 (1%)	1 (2%)	1 (2%)	
Other	4 (3%)	4 (9%)	4 (8%)	
Unknown	6 (4%)	3 (7%)	6 (11%)	
Primary endpoint	63 (40%)	3 (7%)	2 (4%)	< 0.0001

CPR = cardiopulmonary resuscitation, ECLS = extracorporeal life support.

Data are expressed as median (interquartile range) or *n* (%). Kruskal-Wallis test was used.

### Initial VF Group

**Figure 1.**
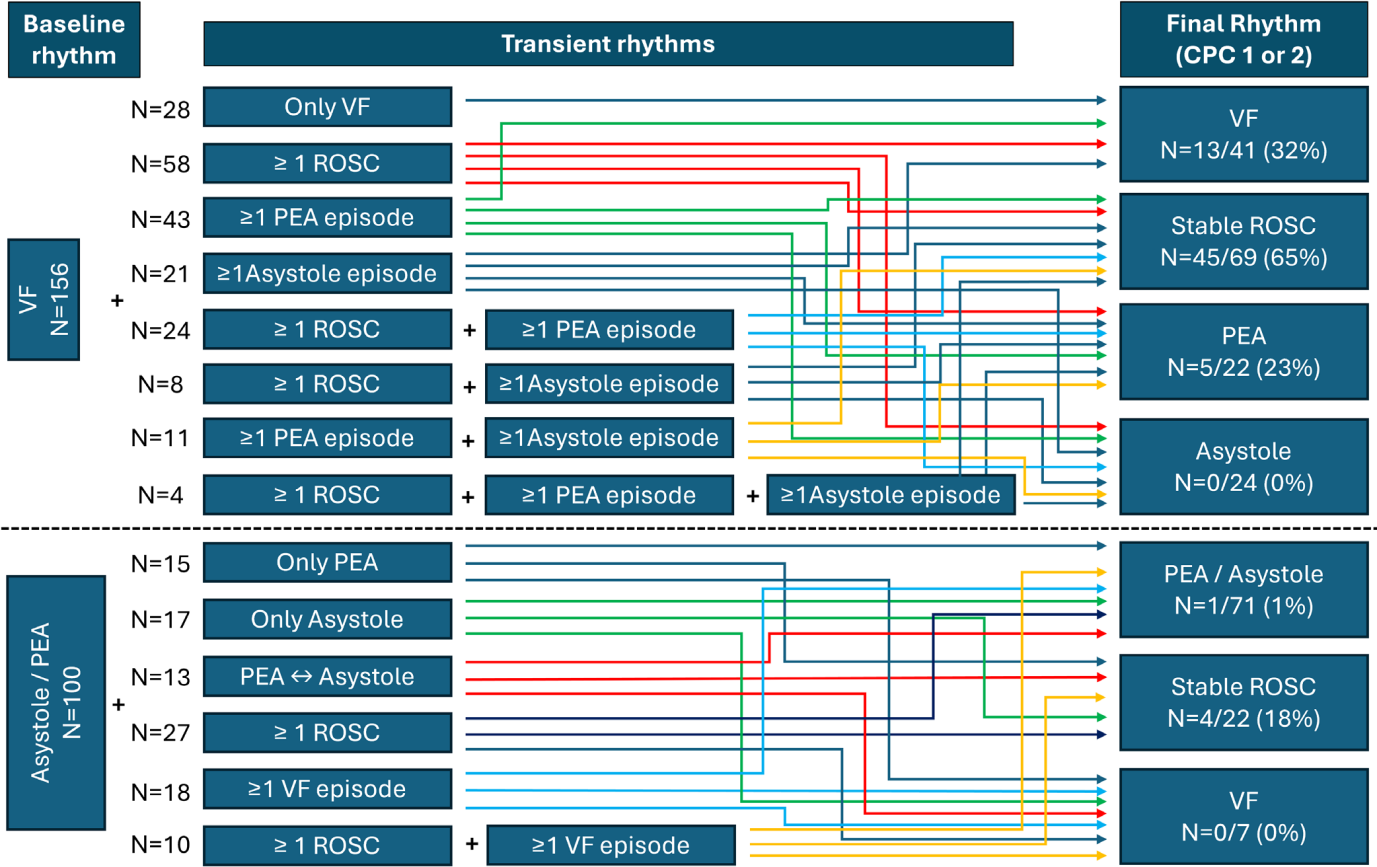
Rhythm profiles in patients with refractory out-of-hospital cardiac arrest during cardiopulmonary resuscitation. Many variants of heart rhythm scenarios with subsequent rhythm conversions could occur during prolonged cardiopulmonary resuscitation. CPC = Cerebral Performance Category, PEA = pulmonary endarterectomy, ROSC = recovery of spontaneous circulation, VF = ventricular fibrillation.

Out of all patients with an initial VF, 28 (18%) had ongoing arrhythmia without any rhythm change, 84 (54%) manifested at least one episode of ROSC, 55 (35%) had at least one episode of PEA, and 39 (25%) converted to at least one episode of asystole. The final rhythm was as follows: VF in 41 (26%), ROSC in 69 (44%), PEA, and asystole in 22 (14%) and 24 (15%) patients, respectively. More details are in **Figure 1**.

Patients with stable ROSC after an initial VF (without any nonshockable rhythm) represented the highest proportion of those achieving 180-day survival at CPC 1 or 2 (32/44 [73%]). When any intermittent episode of a nonshockable rhythm was documented, the survival rates with favorable neurologic outcomes decreased (50/84 [59%]). Patients whose last rhythm was asystole or who had one or more episodes of asystole at any time during CPR had the lowest rate of 180-day survival with CPC 1 or 2 (0/24 [0%] or 5/39 [13%]). For more details, see **Table 2**.

**TABLE 2.** Primary Outcome According to Rhythm Profiles After an Initial Shockable Rhythm

Analysis	Intention to Treat			As Treated		
Treatment Arm	Invasive (*n* = 72)	Standard (*n* = 84)	*p*	Invasive (*n* = 80)	Standard (*n* = 76)	*p*
Ongoing VF (all other rhythms excluded)	7/20 (35%)	1/8 (13%)	0.48	8/22 (36%)	0/6 (0%)	0.2
Last rhythm VF (all intermittent rhythms included)	10/30 (33%)	3/11 (27%)	1.0	13/34 (38%)	0/7 (0%)	0.1
VF + ROSC anytime (nonshockable intermittent rhythms included)	26/34 (76%)	24/50 (48%)	0.02	27/36 (75%)	23/48 (48%)	0.02
VF + ROSC anytime (nonshockable intermittent rhythms excluded)	18/20 (86%)	17/29 (59%)	0.03	19/21 (91%)	16/28 (57%)	0.02
VF + last rhythm ROSC (nonshockable intermittent rhythms included)	21/26 (81%)	24/43 (56%)	0.06	22/28 (79%)	23/41 (56%)	0.09
VF → last rhythm ROSC (nonshockable intermittent rhythms excluded)	15/16 (94%)	17/28 (61%)	0.04	16/17 (94%)	16/27 (59%)	0.02
VF → intermittent ROSC → VF (nonshockable intermittent rhythms excluded)	3/4 (75%)	0/1 (0%)	x	3/4 (75%)	0/1 (0%)	x
VF + PEA anytime (asystole excluded)	7/16 (44%)	8/24 (33%)	0.74	9/19 (47%)	6/21 (29%)	0.37
VF + last rhythm PEA (asystole excluded)	3/6 (50%)	1/11 (9%)	0.2	4/8 (50%)	0/9 (0%)	0.06
VF → intermittent PEA → VF (asystole excluded)	0/5 (0%)	1/1 (100%)	x	1/6 (17%)	0/0 (0%)	x
VF → intermittent PEA → ROSC (asystole excluded)	4/5 (80%)	6/12 (50%)	0.56	4/5 (80%)	6/12 (50%)	0.56
VF → intermittent ROSC → PEA (asystole excluded)	2/3 (67%)	0/1 (0%)	x	2/3 (67%)	0/1 (0%)	x
VF + asystole anytime (PEA included)	3/16 (19%)	2/23 (9%)	0.65	4/18 (22%)	1/21 (5%)	0.25
VF + last rhythm asystole (PEA included)	0/7 (0%)	0/17 (0%)	x	0/7 (0%)	0/17 (0%)	x
VF → intermittent asystole → VF (PEA included)	0/1 (0%)	1/1 (100%)	x	1/2 (50%)	0/0 (0%)	x
VF → intermittent asystole → ROSC (PEA included)	2/5 (40%)	1/3 (33%)	x	2/6 (33%)	1/2 (50%)	x
VF → intermittent asystole → PEA (intermittent ROSC included)	1/3 (33%)	0/2 (0%)	x	1/3 (33%)	0/2 (0%)	x
VF → intermittent ROSC → asystole (PEA included)	0/1 (0%)	0/4 (0%)	x	0/1 (0%)	0/4 (0%)	x

PEA = pulseless electrical activity, ROSC = recovery of spontaneous circulation, VF = ventricular fibrillation.

x—the Fisher exact test cannot be calculated because there were not four values to construct a pivot table.

Data are expressed as *n* (%). The Fisher exact test was used.

In patients who had ROSC at any time during CPR (even though episodes of nonshockable rhythms were documented), the proportion of CPC 1 or 2 at the 180-day follow-up was higher in the ECPR-based/Invasive arm than in the standard arm. Similarly, a higher proportion of CPC 1 or 2 at the 180-day follow-up was documented in patients in the Invasive arm when the last rhythm was ROSC without exhibiting a nonshockable rhythm.

Ten of the 84 patients (12%) with an initial VF allocated to the standard arm crossed over to the invasive strategy. A cross-over from the invasive to the standard arm was done in two of 72 patients (3%) deemed ineligible for advanced invasive therapy. Differences in the primary endpoint rates between treatment strategies were not significantly affected by crossovers. Details are in Table 2.

### Initial Nonshockable Rhythms

A rhythm conversion was documented in 68 patients (68%) with an initially nonshockable rhythm. Conversion of initially nonshockable rhythm to ROSC or VF during CPR was detected in 37 (37%) and 28 (28%) cases, respectively. Stable ROSC was present in 22 patients (22%) at hospital admission. The PEA subgroup had a higher prevalence of intermittent ROSC than the asystole subgroup. Differences in the last rhythm were recognized between patients with initial PEA and asystole. For more details, see **Table 3** and Figure 1.

**TABLE 3.** Rhythm Profiles in Patients With an Initial Nonshockable Rhythm

Initial Rhythm	PEA (*n* = 45)	Asystole (*n* = 55)	*p*
No rhythm change	15 (33%)	17 (31%)	Not significant
Rhythm change	30 (67%)	38 (69%)	
Intermittent rhythm			
ROSC	11 (24%)	4 (7%)	0.008
Asystole^a^	2 (4%)	38 (100%)	
VF	8 (18%)	13 (24%)	
PEA^a^	30 (100%)	2 (4%)	
Last rhythm			
ROSC	6 (13%)	16 (29%)	0.0002
Asystole	12 (27%)	29 (53%)	
VF	5 (11%)	2 (4%)	
PEA	22 (49%)	8 (15%)	

PEA = pulseless electrical activity, ROSC = recovery of spontaneous circulation, VF = ventricular fibrillation.

a The ongoing asystole and PEA were excluded from the calculation.

Data are expressed as *n* (%). The Fisher exact test was used.

Only five patients with a nonshockable initial rhythm survived 180 days with CPC 1 or 2. **Supplemental Table 2** (https://links.lww.com/CCM/H762) shows the profiles of the surviving patients. In two patients, a rhythm change to a shockable rhythm during CPR was documented, constituting two of 28 (7%) of all patients with a conversion from a nonshockable rhythm to VF. Four patients had sustained ROSC at admission (4/22 [18%] of all patients with an initial nonshockable rhythm and sustained ROSC). The last patient, with an initial PEA, did not manifest any rhythm conversion and was treated with venoarterial ECMO (1/35 implanted ECLS in initially nonshockable patients).

### Cox Regression Analysis in the Prediction of Unfavorable Clinical Outcome

In the first Cox regression model (including only variables known up to hospital admission), see **Table 4**, model A, an asystole documented at any time during CPR was independently associated with a higher probability of an unfavorable clinical outcome (the absence of both 180-d survival and a favorable neurologic outcome). Documented ROSC during CPR reduced the probability of an unfavorable clinical outcome. The second Cox regression model, see Table 4, model B, included variables known after the initial in-hospital evaluation (within the first hour of admission). An unfavorable clinical outcome was associated with a CPR length of over 45 minutes, asystole at admission, and the absence of a sustained ROSC at admission when initially nonshockable patients were included.

**TABLE 4.** Cox Regression Analysis in Predicting an Unfavorable Clinical Outcome

Model	Model A (Parameters Available During Ongoing CPR, *n* = 256)		Model B (Parameters After Initial In-Hospital Evaluation, *n* = 156)	
	HR (95% CI)	*p*	HR (95% CI)	*p*
Age ≥ 65 yr	1.09 (0.79–1.5)	0.61	0.94 (0.64–1.37)	0.76
Sex = woman	0.92 (0.64–1.34)	0.68	1.19 (0.77–1.86)	0.43
Telephone assisted bystander CPR = yes	0.91 (0.64–1.28)	0.58	0.84 (0.57–1.23)	0.37
Length CPR > 45 min = yes	—	—	1.85 (1.11–3.08)	0.02
Acute coronary syndrome = yes	—	—	0.92 (0.66–1.3)	0.65
ROSC anytime = yes	0.39 (0.28–0.53)	< 0.0001	—	—
Asystole anytime = yes	2.43 (1.79–3.3)	< 0.0001	—	—
Sustained ROSC at admission = yes	—	—	0.55 (0.32–0.93)	0.02
Asystole at admission = yes	—	—	2.37 (1.53–3.69)	0.0001

CPR = cardiopulmonary resuscitation, HR = hazard ratio, ROSC = recovery of spontaneous circulation.

Tested parameters available during ongoing CPR.

Dashes indicate the parameter was not included to the given model.

## DISCUSSION

This post hoc analysis of the Prague OHCA study contains several novel findings. An invasive strategy may be beneficial in patients with an initial shockable rhythm and intermittent or sustained ROSC. It is more likely in patients with ongoing VF or PEA as the last rhythm. On the contrary, patients with a deterioration of initial VF to asystole seemed to have an inauspicious prognosis despite an invasive approach. However, documentation of VF or sustained ROSC in patients with an initial nonshockable rhythm appears to be a favorable sign for a better outcome. Unfortunately, no evidence exists that an invasive strategy, including ECPR, would improve clinical outcomes in the initial asystole group. The presence of asystole is a significant adverse prognostic marker regardless of the initial rhythm and the phase of initial resuscitation.

### Rhythm Conversions of an Initial VF

Our findings on different outcomes in refractory OHCA for those who presented a transition from a VF to an intermittent or sustained organized rhythm, asystole, or with ongoing VF are in line with previously published data (16, 18), including refractory OHCA populations (19, 20, 24). Bhandari et al (16) presented that a transient organized rhythm had at least twice the survival rate to hospital discharge than patients with ongoing VF or detected asystole. In another study, Kandori et al (18) found in prospective multicenter observational data that the 1-month neurologic outcome of OHCA patients who manifested a conversion to a nonshockable rhythm at hospital admission was very poor compared with patients who had sustained VF. Furthermore, patients with a conversion to PEA had better prognosis than those with asystole (18). However, minimal data elucidates the impact of rhythm changes during CPR on the clinical outcome in refractory OHCA.

Before the Prague OHCA trial, only several studies, mainly retrospective, considered the impact of rhythm changes on patient prognosis in refractory OHCA. Maeda et al (19) showed in a single-center retrospective analysis that conversion to asystole at any time before ECPR in OHCA patients is associated with in-hospital mortality and dismal in-hospital neurologic outcomes. In another study by Nakashima et al (20), patients with a sustained shockable rhythm before the establishment of ECMO support were more likely to have favorable neurologic outcomes at 6 months than those who showed a conversion from an initial shockable rhythm to nonshockable before ECMO initiation. Pozzi et al (28) reported that the occurrence of PEA or asystole at ECMO implantation was associated with a low survival rate and unfavorable neurologic outcomes in refractory OHCA patients. Recent analysis of the ECPR registry showed that heart rhythm at admission is a good predictor of 30-day mortality and neurologic outcomes (24).

The presented post hoc analysis of the Prague OHCA cohort was based on prospectively collected randomized data. Additionally, we enrolled truly refractory patients with prolonged resuscitations reaching 46 and 58 minutes in the standard and invasive arms. Furthermore, some studies refer to rhythm conversion only after initial defibrillation attempts (16, 18). Still, our data analyze the whole duration of the CPR period up to sustained ROSC, ECPR, or declaration of death. Furthermore, the Prague OHCA trial, including this post hoc analysis, compares conventional and invasive (ECPR-based) approaches. Finally, our study reports 180-day survival with a definitively established neurologic status, acknowledging that neurologic recovery can continue to evolve beyond 30 days following OHCA (9). Some previous studies were only focused on ECPR patients and did not evaluate subjects with ROSC (20, 24).

We also show the outcome of VF conversion to asystole separately from PEA. This has been separately analyzed only once in a study by Maeda et al (19). In agreement with their findings, the asystole was associated with higher in-hospital mortality and unfavorable neurologic outcomes in our study. Some previous studies have shown that PEA as the initial rhythm or when deciding to perform ECMO was associated with a trend toward improved survival rates and neurologic outcomes compared with those for asystole (29, 30).

Patients with a rhythm conversion from an initial shockable rhythm to a nonshockable rhythm, especially an asystole, before the initiation of ECPR, did not benefit from this advanced resuscitation strategy. Given that oxygen delivery and myocardial energy substrates might persist in patients with sustained VF, establishing sufficient perfusion of the injured myocardium with ECPR may lead to a higher chance of ROSC and organ recovery. There is also the effect of an invasive strategy on survival with a good neurologic status in patients with intermittent or sustained ROSC. The electrocardiogram rhythm at the presentation and during the CA reflects the etiology and the no-flow or low-flow time (28). If the time from collapse to ECPR onset is prolonged, rhythm conversion to a nonshockable rhythm will occur more frequently (31, 32). We speculate that the rhythm profile could be a better surrogate marker of many less addressable confounders. The invasive approach in the Prague OHCA trial did not only include the ECPR but also included intraarrest mechanical compressions, early transport to a dedicated center, and early invasive evaluation. We hypothesize that all those factors might remain invasive strategy beneficial for OHCA patients even though sustained ROSC is achieved, as suggested in our previous analyses (33), which is contrary to the previously reported deleterious effect of intraarrest transport (34).

### Conversion of Initial Nonshockable Rhythm

The initial nonshockable rhythm appears to be a much better predictor of the clinical outcome than subsequent rhythms in patients suffering from an OHCA. In a study by Cournoyer et al (21), the switch to a shockable rhythm was associated with an improvement in the odds of prehospital ROSC for patients presenting with an asystole. Rhythm conversion, however, did not significantly impact survival. Rajan et al (35) also observed that a conversion to a shockable rhythm was not a more robust prognostic marker than the initial rhythm itself. In a recent meta-analysis, patients who had an initial prehospital asystole experienced ROSC more often after a conversion to a shockable rhythm (22). Other studies confirmed the improved survival and clinical outcomes when subsequent shock delivery is applied (36–39). Goto et al (36) found that subsequent shock delivery in patients with an initial nonshockable rhythm was significantly associated with increased odds of prehospital ROSC, 1-month survival, and 1-month favorable neurologic outcomes when the shock delivery time was less than 20 minutes. It seems that outcomes after OHCA and subsequent shock delivery after an initial nonshockable rhythm may be substantially associated with shock delivery time from collapse (36, 37, 39).

We found that four of five patients with an initial nonshockable rhythm who survived 6 months with CPC 1 and 2 manifested a rhythm conversion to a shockable rhythm or ROSC before in-hospital admission. There was, however, one patient without rhythm conversion who survived while remaining in therapy refractory PEA and applied ECPR as an equivalent to a subsequent shock delivery. Another explanation of rare survivors in a nonshockable group, as reported by Herlitz et al (38), might be misjudged fine VF interpreted as asystole, indicating the possibility of a successful later defibrillation.

Our study did not show the benefit of ECPR in patients with initial nonshockable rhythms. The fact that patients presenting with nonshockable rhythms do not respond to this advanced therapy might be more related to the course and etiology of CA than ECPR itself. We speculate that a shockable rhythm on presentation is a marker for earlier intervention. Indeed, even after conversion to a shockable rhythm, patients having an initial nonshockable rhythm might have more severe underlying total body ischemia concerning the duration of their arrest or the quality of their resuscitation. It is also possible that their underlying illness is less easily treatable than patients with an initial shockable rhythm, regardless of subsequent rhythm conversions.

### Study Limitations

The main limitation of this study was the single-center design, which makes generalization difficult. The data are also limited to regions that have an ECMO center available in a reasonable time. The second limitation was a lack of timing of conversions in the prehospitalization phase. Data was based on medical emergency system staff reports. Third, we cannot rule out that intermittent ROSC was not captured in all cases. Fourth, we cannot rule out that many poorly assessable specifics of the individual OHCA event (i.e., bystander behavior, the effectiveness of CPR, other invasive interventions, etc.) may play a significant role in the outcomes. Finally, the numbers in the analyzed study subgroups are limited, and some crossovers were made. The multicenter study and data on other modalities are sorely needed.

## CONCLUSIONS

The post-initial rhythm profile could more precisely identify an outcome in refractory OHCA patients. Deterioration of the initial shockable rhythm to asystole has a poor prognosis, even when ECPR is readily available. An invasive approach (including intraarrest compressions, early transport, early invasive evaluation, and ECPR) seems beneficial in patients with intermittent or sustained ROSC. It is maybe beneficial in patients with ongoing VF. An initial nonshockable rhythm has an inauspicious prognosis, and a conversion to a shockable rhythm does not seem to improve outcomes.

## ACKNOWLEDGMENTS

We express their immense gratitude to the Prague Emergency Medical Service teams and the Cath Lab and Coronary Care Unit teams of the 2nd Department of Internal Medicine, Cardiovascular Medicine, General University Hospital in Prague, for their inexhaustible efforts in providing superb quality care. Without their continuous commitment, performing this study would not have been possible. We also express thanks to Valerie Reeves for editing the language.

## Supplementary Material


