# Supplementary material for: Cardiac Rhythm Conversions and the Outcome in Refractory Out-of-Hospital Cardiac Arrest: Extracorporeal Versus Conventional Resuscitation
Source: Crit Care Med. 2025 Jul 16;53(10):e1930–40. doi: 10.1097/CCM.0000000000006787 (PMC12490342; doi:10.1097/CCM.0000000000006787)
Supplement: Supplementary file 1 [file ccm-53-e1930-s001.docx]

**Title:**

**CARDIAC RHYTHM CONVERSIONS AND THE OUTCOME IN REFRACTORY OUT-OF-HOSPITAL CARDIAC ARREST. EXTRACORPOREAL VS. CONVENTIONAL RESUSCITATION.**

**Authors:**

Stepan Havranek, M.D., Ph.D., professor ^a^, Jakub Neuhöfer ^a^, Michaela Vesela, M.D. ^a^, Petra Kavalkova, Ph.D. ^a^, Daniel Rob, M.D., Ph.D. ^a^, Zdenka Fingrova, MSc, Ph.D. ^a^, Jana Smalcova, M.D. ^a^, Ondrej Franek, M.D. ^b^, Michal Huptych, MSc., Ph.D. ^c^, Milan Dusik, M.D., Ph.D. ^a^, Jan Pudil, M.D. ^a^, Vojtech Weiss, M.D. ^a^, Ales Linhart, M.D., Ph.D., professor ^a^, Jan Belohlavek, M.D., Ph.D., professor ^a^

**Affiliation:**

^a^ 2^nd^ Department of Medicine – Department of Cardiovascular Medicine, First Faculty of Medicine, Charles University in Prague and General University Hospital in Prague, Czech Republic

^b^ Emergency Medical Service Prague, Czech Republic

^c^ Czech Institute of Informatics, Robotics and Cybernetics (CIIRC), Czech Technical University in Prague, Czech Republic.

**TABLE OF CONTENTS**

Supplemental Table 1…………………………………………………………….. page 2

Supplemental Table 2…………………………………………………………….. page 5

**Supplemental Table 1: Baseline demographical and clinical data.**

| **Initial rhythm** | **VF**  **(n = 156)** | **PEA**  **(n = 45)** | **Asystole**  **(n = 55)** | **P** |
| --- | --- | --- | --- | --- |
| **Age (years)** | 56 (45-64) | 62 (54; 66) | 58 (47; 69) | 0.07 |
| **Gender (Male)** | 141 (90 %) | 31 (69 %) | 40 (73 %) | 0.0003 |
| **Medical history** |  |  |  |  |
| Hypertension | 57/126 (45 %) | 14/26 (54 %) | 18/39 (46 %) | 0.72 |
| Coronary artery disease | 26/125 (21 %) | 2/26 (8 %) | 6/36 (17 %) | 0.28 |
| Chronic heart failure | 10/123 (8 %) | 3/25 (12 %) | 3/37 (8 %) | 0.81 |
| **Bystander CPR** | 154 (99 %) | 44 (98 %) | 54 (98 %) | 0.89 |
| **Time from collapse to EMS arrival (min)** | 9 (6-11) | 8 (6; 10) | 10 (7; 13) | 0.25 |
| **Time from collapse to ACLS (physician arrival) (min)** | 10 (8-13) | 8 (0; 12) | 13 (10; 17) | <0.0001 |
| **Dispatcher assisted CPR** | 133 (85 %) | 24 (53 %) | 46 (84 %) | <0.0001 |
| **Randomized to** |  |  |  |  |
| Standard | 84 (54 %) | 24 (53 %) | 24 (44 %) | 0.41 |
| Invasive | 72 (46 %) | 21 (47 %) | 31 (56 %) |  |
| **Cross over** |  |  |  |  |
| Standard → Invasive | 10/84 | 0/24 | 1/24 | 0.007 |
| Invasive → Standard | 2/72 | 1/21 | 6/31 |  |
| **Time of CPR (time to ROSC or ECLS) (min)** | 54 (33; 69) | 50 (42; 68) | 56 (37; 67) | 0.62 |
| **Admitted to hospital** | 136 (87 %) | 33 (73 %) | 41 (75 %) | 0.03 |
| **Declared death** | 33 (21 %) | 20 (44 %) | 22 (40 %) | 0.0015 |
| Prehospital | 20/33 (61 %) | 12/20 (60 %) | 14/22 (64 %) | 0.009 |
| Within 1 hour of admission | 13/33 (39 %) | 8/20 (40 %) | 8/22 (36 %) | 0.15 |
| **Time to hospital admission (min)** | 55 (46-64) | 46 (38; 58) | 57 (47; 63) | 0.02 |
| **ECLS implanted** | 57 (37 %) | 17 (38 %) | 18 (33 %) | 0.85 |
| **Invasive assessment** |  |  |  |  |
| Coronary angiography | 126/127 (99 %) | 24/25 (96 %) | 31/37 (84 %) |  |
| Pulmonary angiography | 8/127 (6 %) | 10/25 (40 %) | 9/37 (24 %) |  |
| Aortography | 22/127 (17 %) | 8/25 (32 %) | 11/37 (30 %) |  |
| Left ventricle angiography | 29/127 (23 %) | 8/25 (32 %) | 10/37 (27 %) |  |
| **Laboratory values on admission** | | | | |
| pH | 7.00 (6.87-7.17) | 6.85 (6.75; 6.97) | 6.85 (6.77; 6.99) | <0.0001 |
| Lactate (mmol/L) | 10.7 (7.8-13.8) | 13.1 (11.1; 17.0) | 13.9 (8.9; 18.0) | 0.001 |
| **Cause of cardiac arrest (including autopsy findings)** | | | | |
| Acute coronary syndrome | 89 (57 %) | 15 (33 %) | 23 (42 %) | <0.0001 |
| Chronic coronary artery  disease | 29 (19 %) | 1 (2 %) | 2 (4 %) |  |
| Pulmonary embolism | 1 (1 %) | 15 (33 %) | 8 (15 %) |  |
| Chronic heart failure | 8 (5 %) | 2 (4 %) | 4 (7 %) |  |
| Cardiomyopathy | 7 (5 %) | 1 (2 %) | 1 (2 %) |  |
| Myocarditis | 5 (3 %) | 1 (2 %) | 2 (4 %) |  |
| Aortic stenosis | 5 (3 %) | 1 (2 %) | 2 (4 %) |  |
| Aortic dissection type A | 1 (1 %) | 1 (2 %) | 2 (4 %) |  |
| Intracranial hemorrhage | 1 (1 %) | 1 (2 %) | 1 (2 %) |  |
| Bleeding – other | 0 (0 %) | 3 (7 %) | - |  |
| Accidental hypothermia | 3 (2 %) |  | 1 (2 %) |  |
| Pulmonary hypertension | 0 (0 %) |  | 2 (4 %) |  |
| Sepsis | 0 (0 %) | 1 (2 %) |  |  |
| Other | 1 (1 %) | - | 1 (2 %) |  |
| Unknown | 6 (4 %) | 3 (7 %) | 6 (11 %) |  |
| **Primary endpoint** | 63 (40 %) | 3 (7 %) | 2 (4 %) | <0.0001 |

Notes: Data is expressed as median (IQR) or n (%). Kruskal-Wallis’s test was used.

ACLS – Advanced circulatory life support; CPR – Cardiopulmonary resuscitation; ECLS – Extracorporeal life support; EMS – Emergency Medical System; PEA – Pulseless electrical activity; ROSC – Recovery of spontaneous circulation; VF – Ventricular fibrillation.

**Supplemental Table 2: Patients with an initial non-shockable rhythm and CPC 1,2.**

| **Patient No** | **1** | **2** | **3** | **4** | **5** |
| --- | --- | --- | --- | --- | --- |
| **Age (years)** | 35 | 45 | 66 | 58 | 68 |
| **Gender** | Male | Female | Male | Male | Male |
| **Medical history** |  |  |  |  |  |
| Hypertension | No | No | No | No | No |
| Coronary artery disease | No | No | No | No | No |
| Chronic heart failure | Yes | No | No | No | No |
| Diabetes mellitus | Yes | No | No | No | No |
| **Bystander CPR** | Yes | No | No | Yes | Yes |
| **Time from collapse to EMS arrival (min)** | 4 | Collapse after EMS arrival | Collapse after EMS arrival | 10 | 9 |
| **Time from collapse to ACLS (min)** | 8 | 1 | 3 | 17 | 12 |
| **Dispatcher assisted CPR** | No | No | No | Yes | Yes |
| **Randomized to** | Invasive | Invasive | Invasive | Invasive | Standard |
| **Time of CPR** (time to ROSC or ECLS) (min) | 50 | 34 | 49 | 49 | 28 |
| **Initial rhythm** | PEA | PEA | PEA | Asystole | Asystole |
| **Rhythm profile** | PEA  →VF  →PEA  →ROSC | PEA  →ROSC | PEA  →ECLS | Asystole  →VF  →ROSC | Asystole  →ROSC  →BRADY  →ROSC |
| **No of rhythm changes** | 3 | 1 | 0 | 2 | 2 |
| **Ventricular fibrillation** | Yes | No | No | Yes | No |
| **Defibrillation No before admission** | 4 |  |  | 3 |  |
| **Intermittent ROSC** | No | No | No | No | Yes |
| **Sustained ROSC on admission** | Yes | Yes | No | Yes | Yes |
| **Time from EMS arrival to 1^st^ rhythm change (min)** | 5 | 35 |  | 12 | 10 |
| **Time from EMS arrival to 2^nd^ rhythm change (min)** | 13 |  |  | 17 | 25 |
| **Time from EMS arrival to 3^rd^ rhythm change (min)** | 50 |  |  |  |  |
| **Admitted to hospital** |  |  |  |  |  |
| Time to hospital admission (min) | 45 | 34 | 39 | 63 | 61 |
| **ECLS** |  |  |  |  |  |
| ECLS implanted | No | No | Yes | No | No |
| Time to ECLS (min) |  |  | 49 |  |  |
| **Invasive assessment** |  |  |  |  |  |
| Coronary angiography | Yes | Yes | Yes | Yes | Yes |
| Pulmonary angiography | No | Yes | No | Yes | No |
| Aortography | No | No | No | No | Yes |
| Left ventricle angiography | Yes | No | No | No | No |
| **Laboratory values on admission** |  |  |  |  |  |
| pH | 6.921 | 6.998 | 6.826 | 7.188 | 7.204 |
| Lactate (mmol/L) | 11.1 | 12.4 | 14.0 | 8.9 | 5.7 |
| **Cause of cardiac arrest (including autopsy findings)** | Cardiomyopathy | Massive pulmonary embolism | Massive pulmonary embolism + inferior myocardial infarction | Chronic heart failure | Aortic valve stenosis |
| **CPC at 180d** | 1 | 1 | 2 | 1 | 1 |

Note: ACLS – Advanced circulatory support; CPR – Cardiopulmonary resuscitation; ECLS – Extracorporeal circulatory support; EMS – Emergency medical system; PEA
